# Regional overdistension during prone positioning in a patient with acute respiratory failure who was ventilated with a low tidal volume: a case report

**DOI:** 10.1186/s40560-018-0290-z

**Published:** 2018-03-14

**Authors:** Toru Kotani, Masanori Hanaoka, Shinya Hirahara, Hisashi Yamanaka, Eckhard Teschner, Atsuko Shono

**Affiliations:** 1 Department of Anesthesiology and Critical Care Medicine, School of Medicine, Showa University, 1-5-8, Hatanodai, Shinagawa-ku, Tokyo, 142-8666 Japan; 2 Institute of Rheumatology, Tokyo Women’s Medical University, Tokyo, Japan; 3 Draegerwerk AG & Co. KGaA, Luebeck, Germany; 4 Department of Anesthesiology, Shimane University, Izumo, Japan

**Keywords:** Mechanical ventilation, Ventilator-induced lung injury, Prone positioning, Alveolar overdistension

## Abstract

**Background:**

Prone positioning may provide a uniform distribution of transpulmonary pressure and contribute to prevent ventilator-induced lung injury. However, despite moderate positive end-expiratory pressure and low tidal volumes, there is still a risk of regional overdistension.

**Case presentation:**

A man with refractory hypoxemia was mechanically ventilated with prone positioning. Although prone positioning with a plateau pressure of 18 cmH_2_O and a positive end-expiratory pressure of 8 cmH_2_O promptly improved oxygenation, regional ventilation monitoring using electrical impedance tomography initially detected decreased distribution in the dorsal region but increased in the ventral, suggesting overdistension.

**Conclusions:**

Our experience indicates monitoring regional ventilation distribution is useful for decreasing the risk of overdistension during prone positioning.

## Background

Inappropriate settings during mechanical ventilation can induce or exaggerate uneven distribution of ventilation, cause multiple organ dysfunction (ventilator-induced lung injury (VILI)), and increase mortality of patients with acute respiratory distress syndrome (ARDS) [1]. Prone positioning may provide a more uniform distribution of transpulmonary pressure [2]. This, at least partly, may contribute to preventing VILI because of uneven distribution. In a recent study, prolonged prone positioning combined with a low tidal volume strategy decreased the mortality of ARDS [3]. However, lung protection of the dorsal region is not guaranteed during prone positioning. Although the ventilator settings that are applied during prone positioning may be different from those during the supine position in patients with ARDS, they have not been systematically investigated. A risk of overdistension of the dorsal regions still exists in some conditions.

We experienced a case of lung overdistension in the dorsal region during prone positioning as assessed by electrical impedance tomography in a patient with ARDS. Lung overdistension occurred, despite the fact that the patient was ventilated according to the lung protective concept. We report the details of this case with a literature review.

## Case presentation

A 79-year-old man presented with dyspnea and hypoxemia. He had rheumatoid arthritis and received immunosuppressants for several years. Before admission, he had a cough and fever for 3 days. Arterial blood oxygen saturation measured by pulseoxymeter (SpO_2_) was 94% with standard oxygen therapy, and a chest radiograph showed bilateral ground glass opacity. Because oxygenation became increasingly worse, he was transferred to the intensive care unit. Non-invasive positive pressure ventilation (BiPAP Vision; Fuji-Respironics, Tokyo, Japan) was started immediately. We performed bronchoalveolar lavage, and *Pneumocystis jirovecii* pneumonia was confirmed by laboratory testing. Intravenous administration of a combination of trimethoprim (15 mg/kg) and sulfamethoxazole (75 mg/kg) plus methylprednisolone was started.

**Fig. 1:**
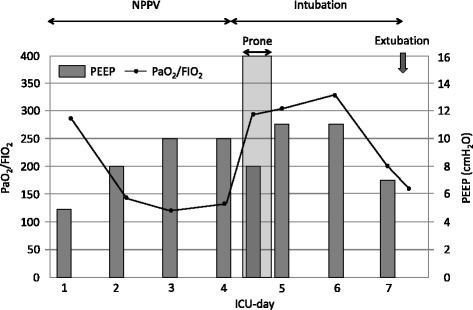
Mechanical ventilation and oxygenation before, during, and after prone positioning

Because the patient received long-term steroid therapy, we initially planned to avoid tracheal intubation for preventing ventilator-associated complications. Oxygenation was maintained with a positive end-expiratory pressure (PEEP) of 8 cmH_2_O and a fraction of inspired oxygen (FIO_2_) of 0.45 in the first 3 days. On day 4, SpO_2_ was frequently below 88%, and hypoxemia was refractory to elevation of FIO_2_ (0.6 to 0.8). We started bedside online monitoring of the distribution of regional ventilation using electrical impedance tomography (EIT; PulmoVista 500, Draegerwerk, Luebeck, Germany) to titrate ventilation pressure. After PEEP was increased from 8 to 10 cmH_2_O, dynamic imaging of EIT showed no improvement in the distribution of ventilation, and PaO_2_/FIO_2_ progressively decreased to less than 150 mmHg (Fig. 1). The patient was intubated and ventilated with a PEEP of 8 cmH_2_O. A plateau pressure was initially set at 21 cmH_2_O, but decreased to 18 cmH_2_O to adjust for a tidal volume of 6 to 7 ml/kg (predicted body weight) as a lung protective setting and to obtain uniform distribution on EIT. However, the dorsal region was still poorly ventilated and oxygenation was not improved. Therefore, we decided to perform prone positioning with the same ventilation settings.

**Fig. 2:**
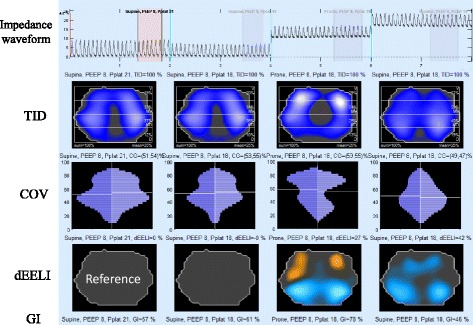
Impedance waveform, tidal images (TID), center of ventilation (COV), change of end-expiratory lung impedance against reference (dEELI), and global inhomogeneity index (GI) before, during, and after prone positioning. In TID, the white area indicates being maximally ventilated. In dEELI, orange or blue color indicates the decrease or increase of impedance value compared to the reference (supine, plateau pressure of 21 cmH_2_O), respectively. Ventral shift of ventilation was unexpectedly observed during prone positioning

The tidal images of ventilation distribution (TID) before, during, and after prone positioning are presented in Fig. 2. After prone positioning was started, PaO_2_/FIO_2_ increased to 296 mmHg without any hemodynamic changes. Interestingly, TID clearly showed the distribution of ventilation was initially not shifted to the dorsal region, but to the ventral region (white area in TID, prone). Because dynamic images of EIT and SpO_2_ showed no further change, we terminated prone positioning after 4 h. After prone positioning was terminated, the ventral shift disappeared and homogeneous distribution was obtained with the same ventilation settings and no adverse effects from prone positioning were found. No serious deterioration of gas exchange was observed after the prone positioning. Oxygenation was stabilized with a PEEP of 11 cmH_2_O in the next 2 days. On day 7, PEEP was incrementally decreased to 7 cmH_2_O and the patient was successfully weaned from mechanical ventilation (Fig. 1). The patient was discharged from the intensive care unit on day 11 and the hospital on day 58.

We performed offline analysis of the saved impedance data using software (EIT diag; Draegerwerk) for further analysis using different parameters, center of ventilation (COV) [4] and global inhomogeneity index (GI) [5], and by quantifying the results of online monitoring. COV (presented as CG in Fig. 2) describes the distribution of tidal ventilation along the gravitational axis. When the location of the center of ventilation is expressed as a percentage of the dorsal-to-ventral thorax diameter, a value of 50% represents equal distribution between the ventral and dorsal regions. GI is quantified as the standard deviation of the proportion of total tidal impedance variation. A smaller GI index delineates more homogeneous distribution in tidal ventilation. Figure 2 shows the results of the analysis. Lung volume, represented by end-expiratory lung impedance (EELI), did not change when a plateau pressure was decreased (top, left to mid-left), and was significantly increased over time after prone positioning was started (top, mid-left to mid-right). TID showed a more homogeneous and normal distribution before prone positioning, and ventral shift of ventilation was clearly detected during prone position (mid-right). Interestingly, dEELI (change in EELI at different time point) images showed that the dorsal lung volume was significantly increased (colored blue) despite the same ventilator settings, as a result of prone positioning. Distribution of ventilation in each region of interest (ventral, md-ventral, mid-dorsal, and dorsal) was shown as a percentage of total ventilation in TID (on the right side of each graph). Ventilation distribution initially decreased from 36 to 25% in the mid-dorsal region and increased in the ventral region to the same extent (20 to 29%). Additionally, ventral shift of COV was observed from 53 to 59% in the right lung. After prone positioning, distribution of ventilation in the mid-ventral and ventral regions decreased from 63 to 41%, and the values were maintained in the supine position until the next day.

## Discussion

Animal [6, 7] and clinical investigations [8–12] have reported that regional ventilation monitoring during prone positioning using the parameters of EIT, including TIV, COV, GI, and dEELI, play a pivotal role in pressure titration of mechanical ventilation.

In our case, EIT initially detected less ventilated area in the dorsal lungs. Poor oxygenation under higher ventilation pressures met the indication criteria for prone positioning. Prone positioning was suitable to facilitate uniform ventilation to prevent VILI rather than to recruit the lungs. Gas exchange was promptly improved during prone positioning as we expected, while a significant increase of dorsal lung volume (dEELI in Fig. 2) was observed at the same time. EIT is useful to assess the recruitment effect of prone positioning. However, dorsal recruitment and improvement of the ventral/dorsal balance (COV) and homogeneity (GI) were not obtained during, but only after, prone positioning. The significant ventral shift of ventilation (in the ventral region from 20 to 29%) was unexpected during prone positioning. This phenomenon needs further explanation because dorsal recruitment is typically associated with a dorsal shift in distribution of ventilation [12]. One possible explanation is that proning the patient changes the three-dimensional shape of the diaphragm and thus eliminates the “sand-bag” effect of the abdominal compartment on the dorsal lung. Consequently, a significantly lower PEEP might have been required to counterbalance the abdominal pressure. However, as the applied pressure remained unchanged during the phase of prone positioning, it became excessive at some time point and caused overdistension in the dorsal half of the lung. This explanation is supported by the significant increase in lung volume in the whole dorsal region during prone positioning as indicated by the dEELI image (Fig. 2). This massive increase of lung volume caused overdistension and impeded distribution of gas in the dorsal regions. The possibility that the change in impedance value was caused by a change in skin electrode impedance could be excluded by verifying that the skin-electrode transfer impedance between the pre-prone, prone, and post-prone phases remained in the same range. However, the possibility that the three-dimensional reshaping of the diaphragm may also have partly contributed to the observed change in ventilation distribution, especially in the mid-ventral and mid-dorsal regions, cannot be completely excluded. During prone positioning, TID revealed a large non-ventilated area, which might have been caused by a caudal-cranial shift of the mediastinum. However, when comparing the status before and during the prone positioning, the dorsal half of the TID and dEELI images present the typical patterns of overdistension: the concurrent decrease of ventilation and increase of lung volume, respectively.

It is well known that the distribution of ventilation is affected by gravity and thus prone positioning has been used to obtain homogeneous distribution of ventilation [13]. Theoretically, overdistension can occur in the prone position if the ventilation pressure is higher than the level each lung condition requires. To the best of our knowledge, this is the first report to detect a condition where prone positioning has caused regional overdistension. Notably, ventilation pressure was moderate, but still caused overdistension in this case.

Our results raise two important issues. First, there is no ideal number for safety in ventilator settings. In mechanical ventilation, the lowest, but effective, value is preferred to prevent ventilator-associated events. Although oxygenation was improved and eventually our ventilation settings led to successful results, the data suggest that a lower PEEP was able to improve the oxygenation with a lower risk of overdistension. EIT is a useful measure to determine this value at the bedside. Second, the effect of a specific pressure can be different in the prone position compared with the supine position. In fact, PEEP was elevated from 8 to 11 cmH_2_O after prone positioning to maintain regional ventilation and oxygenation in the current case. The threshold of ventilation pressure has only been studied in the supine position. Our results suggest that in the case presented in this report, ventilation pressure should have been set lower in the prone position than in the supine position.

Regional overdistension can be detected by limited measures. Analysis using computed tomography is a potential method, but its clinical use is limited because of inconvenience of frequent examinations owing to radiation exposure and the risk of transportation. The analysis is only static. EIT is another method of detecting regional overdistension, but the monitoring area is limited. We did not perform chest-computed tomography in our case. The incidence and clinical effect of prone positioning-associated overdistension are unclear. We did not find any further cases of suspected prone positioning-associated overdistension in our cohort. Our patient had been receiving long-term steroid therapy, and that could have contributed to developing overdistension with a relatively low ventilation pressure. Further studies are needed to investigate the hazardous effects of dorsal overdistension on clinical outcomes. In contrast to computed tomography, where Gattinoni et al. once defined a voxel with less than − 900 HU as being overdistended [14], EIT does not have an established absolute threshold to diagnose overdistension. This is a limitation of an EIT-guided method.

## Conclusion

In conclusion, regional overdistension was detected by analysis of EIT during prone positioning, despite performing lung protective ventilation in a patient with acute respiratory distress syndrome. Our experience suggests that dorsal overdistension can occur during prone positioning and regional monitoring to decrease the risk of overdistension is warranted.

## Abbreviations

ARDS: Acute respiratory distress syndrome
COV: Center of ventilation
dEELI: Changes of EELI
EELI: End-expiratory lung impedance
EIT: Electrical impedance tomography
FIO_2_: Fraction of inspired oxygen
GI: Global inhomogeneity index
PEEP: Positive end-expiratory pressure
SpO_2_: Arterial blood oxygen saturation measured by pulseoxymeter
TID: Tidal images of ventilation distribution
VILI: Ventilator-induced lung injury
